# Exertion during a hypoxia altitude simulation test helps identify potential cardiac decompensation

**DOI:** 10.1002/rcr2.450

**Published:** 2019-06-24

**Authors:** Leigh Seccombe, Matthew Peters, Claude Farah

**Affiliations:** ^1^ Thoracic Medicine Concord Hospital Sydney New South Wales Australia; ^2^ Faculty of Medicine and Health Sydney University Sydney New South Wales Australia

**Keywords:** Clinical respiratory medicine, environmental and occupational health and epidemiology, pulmonary circulation and pulmonary hypertension, respiratory function tests, respiratory structure and function

## Abstract

A 64‐year‐old female with a history of chronic thromboembolic pulmonary arterial hypertension (CTEPH), moderate airway obstruction (forced expiratory volume in 1 second (FEV_1_) 58% predicted), and resting oxygen saturation below the normal range (SaO_2_ 94%) underwent a hypoxic challenge test (HCT) to determine suitability for long‐haul air travel. The HCT showed only a mild decrease in SaO_2_ (89% at 0.15 fraction of inspired oxygen (FIO_2_)) at rest. However, a HCT coupled with mild exercise at two metabolic equivalents demonstrated significant hypoxia (SpO_2_ 77%) with worsening right ventricular impairment and an inability to increase cardiac output measured with echocardiography. The case highlights the importance of the evaluating cardiac and pulmonary reserve during hypoxic stress. Resting measures alone may not identify risk, and the addition of an exercise component was essential in this case.

## Introduction

A 64‐year‐old female with a history of chronic thromboembolic pulmonary arterial hypertension (CTEPH) and bronchiectasis requested medical assessment regarding an impending holiday to Hawaii. Travel included a 10‐h return commercial aircraft flight with intent to visit lowland areas only. Her medications included bosentan and sildenafil, and her most recent respiratory function test demonstrated moderate airway obstruction (forced expiratory volume in 1 second (FEV_1_) 58% predicted 1) with normal vital capacity (91% predicted 1). Arterial saturation (SaO_2_) on room air was 94%, and pressure of arterial oxygen (PaO_2_) was 65 mmHg. She had not recently travelled by commercial aircraft.

## Case Report

A hypoxic challenge test (HCT) was performed to determine the suitability of supplemental oxygen in‐flight as recommended for patients with comorbidities that are worsened by hypoxemia 2. The HCT assesses the patient's response to breathing a reduced fraction of inspired oxygen (FIO_2_) of 0.15 for 20 min, replicating a pressure of inspired oxygen under worse‐case cabin pressure conditions of 574 mmHg 3. Patients that experience a PaO_2_ < 50 mmHg or pulse oximetry (SpO_2_) <85% require supplemental oxygen in‐flight 2.

Prior to testing, the patient gave written informed consent to include echocardiography 4, 5 (GE EchoPac 7.0.1, Horten, Norway) during the HCT both at rest (one metabolic equivalent) and during mild exercise equivalent to <3 km/h, level ground walking (two metabolic equivalents or ≈7–8 mL/min/kg VO_2_) (Oxycon Pro, Jaeger‐Toennies, Hochberg, Germany), as part of a research study investigating haemodynamic responses in pulmonary hypertension (HREC/14/CRGH/198; Human Ethics Review Board, Sydney Local Area Health District, New South Wales, Australia).

In this case, the HCT following a standard protocol at rest induced a fall in PaO_2_ to 54 mmHg and SaO_2_ to 89%, indicating that supplemental oxygen in‐flight was not necessary according to consensus guidelines. However, significant hypoxia developed (SpO_2_ 77%) when the HCT was performed with the addition of mild exercise at two metabolic equivalents (7.3 mL/min/kg VO_2_), the mean heart rate at rest on room air was 89 bpm and increased during the HCT to 94 bpm at rest and to 110 bpm with exercise. The patient did not report breathlessness at any stage of the study (Borg scale 0 (0–10)).

**Figure 1:**
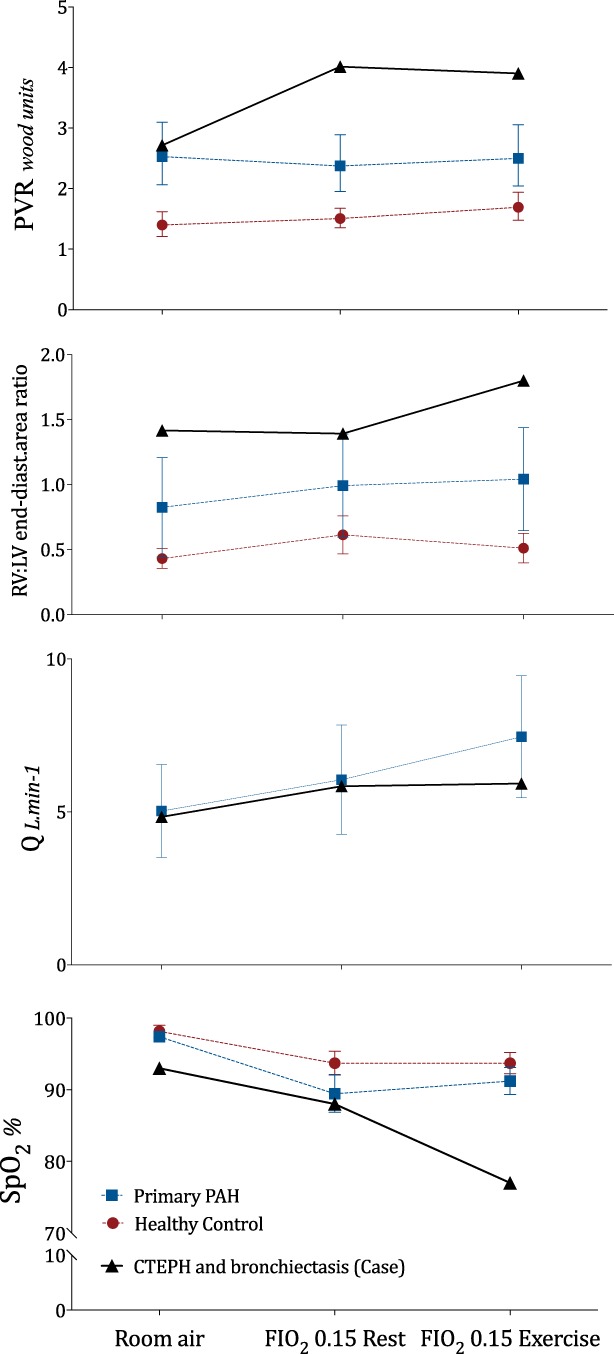
The case subject's response to a hypoxic challenge test (FIO_2_ 0.15) at rest and with mild exercise in comparison to patients with primary pulmonary hypertension (PAH) and healthy controls 6. FIO_2_, fraction of inspired oxygen; LV, left ventricular; PAH, pulmonary arterial hypertension; PVR, pulmonary vascular resistance; Q, cardiac output; RV, right ventricular; SpO_2_, pulse oximetry.

Echocardiography demonstrated an increase in pulmonary vascular resistance (PVR) in parallel with right ventricular (RV) impairment (increased right: left ventricular (LV) end diastolic area ratio) and a non‐changing cardiac output (Q). Figure 1 shows this case subject's HCT results (solid line) in comparison to data collected from subjects with primary pulmonary hypertension (dotted line, square) and healthy normal subjects (dotted line, circles) 6.

## Discussion

This case clearly illustrates the interdependence of the cardiac and ventilatory responses to exercise, especially in a hypoxic situation. The indication for the HCT in this case was based on the patient's known CTEPH and oxygen saturation below the normal range on room air in line with clinical practice, whereby an HCT forms part of the “fit‐to‐fly” clinical assessment. The addition of mild exercise along with the HCT unveiled the propensity for the patient to develop hypoxia and worsening RV dysfunction but, interestingly, without reported symptoms. The case highlights the limitation of the HCT performed only at rest and reinforces the importance of evaluating a patient's cardiac, as well as pulmonary, reserve when assessing patients’ fitness for long‐haul air travel.

Patients with primary pulmonary hypertension have been shown to mitigate the effects of lower FIO_2_/PaO_2_ during an HCT via increased Q without any further increase in PVR or an appreciable change in RV chamber size or function (Fig. 1) 6. However, in this subject with particularly raised right: LV end diastolic ratio at rest and moderate airways disease, PVR worsened during the HCT in resting conditions, while oxygenation remained “acceptable.” However, significant oxygen desaturation developed once the demand for oxygen increased via mild exercise. In this case, testing was terminated, and in‐flight supplemental oxygen was recommended for her flight to Hawaii. The lack of a further increase in Q with exercise in this case report is unexpected and implies a reduction in stroke volume as the heart rate increased with exercise as expected. RV dysfunction is demonstrated with a further increase in the RV:LV ratio that may have developed due to pressure overload. The mechanisms that link these significant haemodynamic changes with oxygen desaturation are likely to be complex. Patients with severe airways disease desaturate when exposed to an HCT, but this is unlikely to be the sole cause in this case as the patient's bronchiectasis was not severe. An alternative mechanism could relate to RV dilatation and ventricular interdependence, resulting in right‐to‐left shunting and thus accentuating the hypoxaemia. Another possibility may be due to subclinical pulmonary oedema and increased LV preload developing as a consequence of the RV dilatation.

Air travel is generally very safe. Symptoms are commonly reported in those with pulmonary arterial hypertension 7 and chronic respiratory disease 8; however, related adverse medical events are infrequent 8. Significant, stable hypoxemia is tolerated well, with compensatory mechanisms maintaining the required oxygen uptake.

Identifying those at risk where compensatory mechanisms become exhausted, such as in this case, is challenging. The HCT offers poor risk stratification considering the primary (only) end‐point is oxygen saturation. In this case, echocardiography provided important insight on the underlying mechanisms of limitation, with a strong risk of decompensation suggested with any further exposure to hypoxia or exercise stress.

Recommendations state that an FEV_1_ > 30% predicted, HCT PaO_2_ > 50 mmHg, and Functional Class I‐II for pulmonary hypertension 2 present a low risk of a medically adverse event in‐flight. While the case subject met these criteria, comorbidity has confounded tolerance to hypoxia. The HCT has been questioned with regard to its clinical relevance 2, 9. It is possible that the addition of haemodynamic parameters, as well as some form of exertion during the HCT, could provide more clinically relevant information. Such a modified protocol would need validation in a larger study and would provide an opportunity for further exploration of the exact mechanisms that link cardiac and pulmonary factors causing oxygen desaturation in patients with comorbid disease.

### Disclosure Statement

Appropriate written informed consent was obtained for publication of this case. This case data were part of a study approved by Human Ethics Review Board, Sydney Local Area Health District, New South Wales, Australia (HREC/14/CRGH/198). Permission from the Sydney Local Health District Human Ethics Review Board (NSW, Australia) was obtained by the authors to access and publish group data (patients with pulmonary hypertension and healthy controls) collected under the same approved ethics application.
